# ﻿On the type specimens of representatives of the subgenus Apterosoma Motschulsky, 1861 of the genus *Chrysolina* Motschulsky, 1861 (Coleoptera, Chrysomelidae)

**DOI:** 10.3897/zookeys.1252.146087

**Published:** 2025-09-19

**Authors:** Takuya Takemoto, Andrzej O. Bieńkowski, Satoru Saitoh

**Affiliations:** 1 Systematic Entomology, Graduate School of Agriculture, Hokkaido University, Sapporo-shi 060-8589, Japan Hokkaido University Sapporo-shi Japan; 2 Zelenograd, 1131-165, Moscow 124460, Russia Unaffiliated Moscow Russia; 3 Overseas Department, Tonichi Engineering Consultants, INC, 1-13-3, Honmachi, Shibuya-ku, Tokyo 151-0071, Japan Tonichi Engineering Consultants Tokyo Japan

**Keywords:** *
Apterosoma
*, *
Chrysolina
*, Chrysomelidae, Chrysomelinae, Coleoptera, Japan, Palaearctic, taxonomy, type

## Abstract

Classification of the subgenus Apterosoma Motschulsky of the genus *Chrysolina* is fraught with several problems. In particular, the northern Japanese *Apterosoma* can be separated into at least 20 morphologically and/or genetically distinct, yet undescribed, “units”. In this study, we examine type specimens of the three species included within the subgenus to determine their relationships to these units and to each other.

## ﻿Introduction

The subgenus Apterosoma Motschulsky, 1861 consists of three species, *Chrysolina angusticollis* (Motschulsky, 1861), *Chrysolina aino* Takizawa, 1970 and *Chrysolina porosirensis* Takizawa, 1970 (Kippenberg and Mikhailov 2024). After Takizawa (1970) described *Ch. aino* and *Ch. porosirensis*, Hasegawa (1980) pointed out that the morphological characters used by Takizawa to distinguish *Ch. aino* from *Ch. angusticollis* exhibited regional variation, and therefore, the taxonomic distinction between the two species should be reexamined.

Hasegawa (1980) was the first to confirm the presence of intraspecific variation within these species and examined two external characters not considered by Takizawa (1970): the structure of the hind wings and the sculpture of the elytral surface. As a result, he confirmed clear differences between *Ch. angusticollis* and *Ch. aino*. He found that in *Ch. angusticollis*, the hind wings tend to be more reduced in more southern populations, while in *Ch. aino* and *Ch. porosirensis*, the hind wings are even more strongly reduced. He also found that *Ch. angusticollis* is characterized by a reticulate, coriaceous sculpture on the elytral surface, which is especially conspicuous in females and tends to be denser and more prominent in northern populations, whereas *Ch. aino* and *Ch. porosirensis* lack this feature in both sexes. After that, these three species have been treated as part of the “units” within the northern Japanese *Ch. angusticollis* species complex (= northern Japanese *Apterosoma*), which can be divided into at least 20 morphologically and/or genetically differentiated groups (Saitoh 2012). Because these units currently lack taxonomic treatment, they require appropriate study to determine their validity as separate species or subspecies.

According to Saitoh et al. (2008) and Saitoh (2010, 2012), the population treated as *Ch. angusticollis* by Takizawa (1970) corresponds to morphological groups A and B: Units A-1, A-2, B-1 and B-2; and the populations treated by Hasegawa (1980) correspond to morphological groups A, B and C: Units A-1, A-2, B-1, B-2, C-1, C-2 and C-3. The population treated as *Ch. aino* by Takizawa (1970) corresponds to morphological group EIII: Units EIII-1, EIII-2; and the population studied by Hasegawa (1980) corresponds to morphological groups EII and EIII: EII-1, EII-2, EIII-1 and EIII-2. The population treated as *Ch. porosirensis* by Takizawa (1970) and Hasegawa (1980) corresponds to morphological group F: Unit F (Table 1). None of the above researchers were able to check the type specimen of *Ch. angusticollis*, and Motschulsky recorded only “JAPAN” as the type locality in the original description, so it is not certain which units comprise the “true” *Ch. angusticollis*. As discussed in Saitoh (2010), although each unit can be partially distinguished based on external morphology, their phylogenetic relationships remain unresolved. Therefore, each unit should be classified as an independent species or subspecies based on future genetic analyses.

**Table 1.** A correspondence table between the Morphological Groups and Units established by Saitoh (2012) and the subgenus Apterosoma as defined by Motschulsky (1861), Takizawa (1970), and Hasegawa (1980) (Saitoh 2012, revised).

Morphological Group	Unit	Motschulsky, 1861	Takizawa, 1970	Hasegawa, 1980
A	A-1	–	*Ch. angusticollis*	*Ch. angusticollis*
	A-2	–	*Ch. angusticollis*	*Ch. angusticollis*
B	B-1	–	*Ch. angusticollis*	*Ch. angusticollis*
	B-2	–	*Ch. angusticollis*	*Ch. angusticollis*
C	C-1	*Ch. angusticollis*	–	*Ch. angusticollis*
	C-2	–	–	*Ch. angusticollis*
	C-3	–	–	*Ch. angusticollis*
D	D	–	–	–
EI	E1-1	–	–	–
	E1-2	–	–	–
EII	EII-1	–	–	*Ch. aino*
	EII-2	–	–	*Ch. aino*
	EII-3	–	–	*Ch. aino*
	EII-4	–	–	*Ch. aino*
	EII-5	–	–	*Ch. aino*
EIII	EIII-1	–	*Ch. aino*	*Ch. aino*
	EIII-2	–	*Ch. aino*	*Ch. aino*
F	F	–	*Ch. porosirensis*	*Ch. porosirensis*
G	G	–	–	–
H	H	–	–	–

In this study, to ensure proper taxonomic treatment of the units in the *Ch. angusticollis* species complex, we checked one of the syntypes of *Ch. angusticollis* and the types of the other two species of the subgenus Apterosoma, and considered how they should be classified in relation to the three existing species concepts.

## ﻿Material and methods

The type specimens examined herein are preserved in the following museums: Zoological Museum of Moscow State University, Moscow, Russia (ZMMU); Natural History Museum, London, United Kingdom (BMNH); and Systematic Entomology Collection, Hokkaido University, Sapporo, Japan (SEHU). The additional specimens are preserved in the private collection of Takuya Takemoto (TTPC).

**Figure 1.**
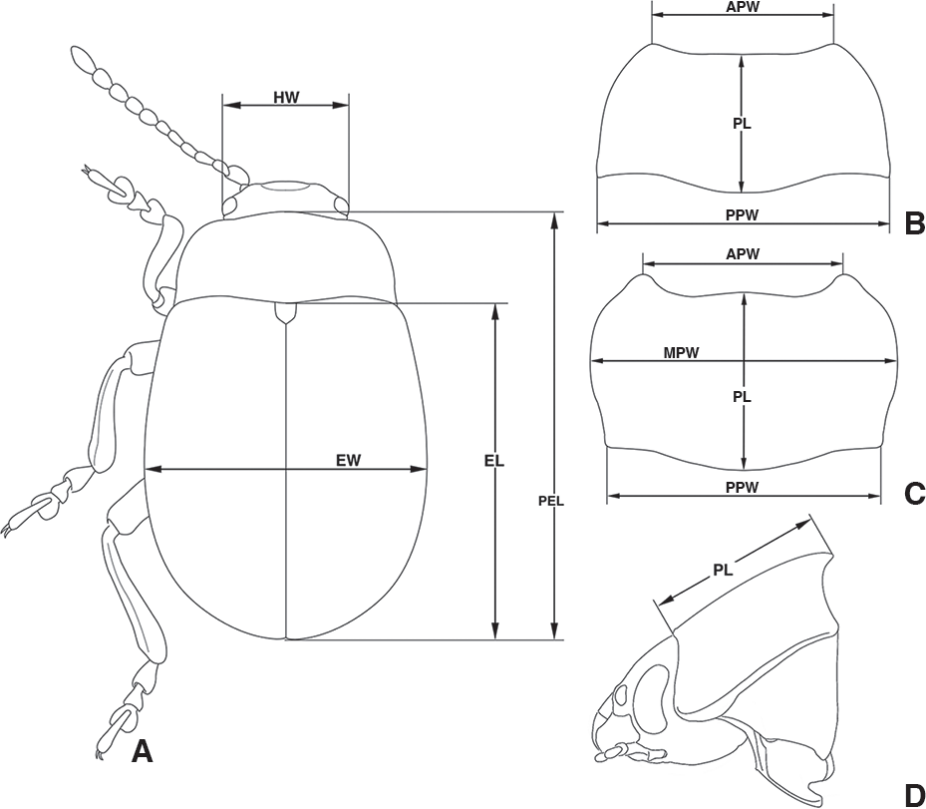
Parts for biomimetic measurements (Takemoto 2022, revised). **A.** Habitus, dorsal view; **B.** Pronotum of species in dorsal view (GWBP ― group with pronotum widest at base); **C.** Pronotum of species in dorsal view (GWCP ― group with pronotum widest at the center of the lateral margin); **D.** Head and pronotum in lateral view. Abbreviations: HW ― width of head including eyes; PL ― length of pronotum; APW ― pronotum width at anterior margin; MPW ― maximum pronotum width; PPW ― width of posterior margin of pronotum; EL ― length of elytra measured from top of scutellum to apex of each elytron; EW ― maximum width across elytra.

To observe male genitalia, specimens were relaxed in boiling water for 15 minutes before removing the abdomen from the body. Genitalia were then removed from the abdomen and softened in KOH solution (c.10%) for 15 minutes at 60 °C before removing muscle tissue from the genitalia in ethanol (c. 80%). Genitalia were then dyed in a solution of lactic acid and acid fuchsine for 3 h at 60 °C, then dehydrated in acetic salicylate for 15 min at 6 °C, and then in xylene for 2 min at ambient temperature. Genitalia and larvae were observed in α-terpineol using a stereo microscope (Nikon SMZ745T, SMZ800). All photos were edited using Adobe Photoshop 2024. Abbreviations for measurements follow Takemoto (2022), with the addition of MPW (maximum pronotum width), to account for groups such as the subgenus Apterosoma of genus *Chrysolina* in which the pronotum is widest at the middle (Fig. 1C). We are referring to the groups as GWCP (group widest at the center of the lateral margin of the pronotum) (Fig. 1C) and GWBP (group widest at base of pronotum) (Fig. 1B).

Exact label data are cited for all type specimens of described species; a double slash (//) separates different labels, and a single slash (/) divides the different rows of data on a label.

## ﻿Results

**Genus *Chrysolina* Motschulsky, 1861**

### ﻿Subgenus Apterosoma

Taxon classificationAnimaliaColeopteraChrysomelidae

Motschulsky, 1861

C2F11D9F-B507-5093-8FEC-68BD00BEA2A3

Apterosoma Motschulsky, 1861: 23 [type species: Apterosoma angusticollis Motschulsky, by monotypy].
Caudatochrysa Bechyné, 1950: 149 [type species: Apterosoma angusticollis Motschulsky]; Bieńkowski (2001) synonymized with Apterosoma.

### ﻿ Chrysolina angusticollis

Taxon classificationAnimaliaColeopteraChrysomelidae

(Motschulsky, 1861)

83B50CA0-25D2-5732-97E0-D7319F3E7408

Figs 2, 3A–J, 4

Apterosoma angusticollis Motschulsky, 1861: 23 (Japan, syntype in ZMMU).
Chrysomela japana Baly, 1874: 171 (Hakodadi, Japan, syntypes in BMNH).

#### Type material examined.

***Syntype*** • 1 ♀ (ZMMU), [Yellow card with no text] // Type // Apterosoma / angusticollis / Motch / Japan. [typed on a yellow card].

**Figure 2.**
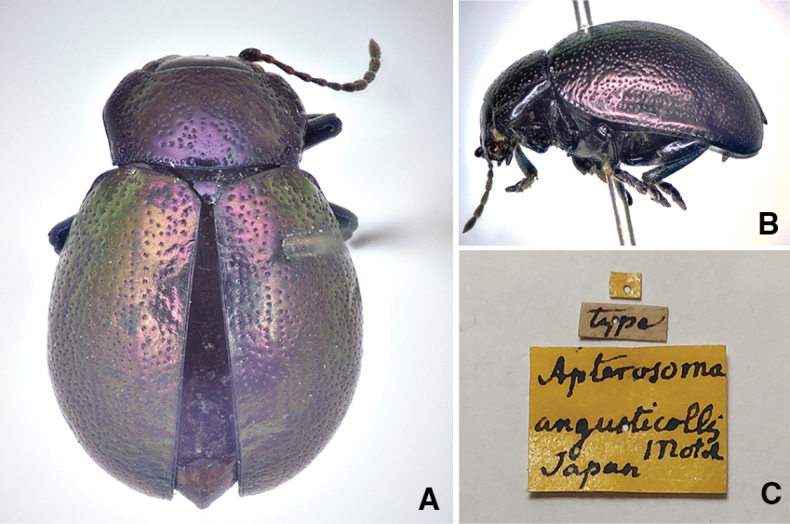
*Chrysolina angusticollis* (Motschulsky, 1861), syntype. **A.** habitus, dorsal view **B.** habitus, lateral view **C.** labels. Photographs by Andrzej O. Bieńkowski.

#### Measurements of syntype.

HW 2.53 mm; PEL 8.76 mm; EL 6.86 mm; EW 5.70 mm; APW 2.74 mm; MPW 3.91 mm; PPW 3.69 mm; PL 2.43 mm.

#### Additional material examined.

**Japan.** Hokkaido: • 1 ♀ (SEHU), Mt. Yokotsu, Mt. Eboshi, near Hakodate, 19. IX. 1959, K. Munakata leg., MUNAKATA Coll., SEHU JAPAN, 2019, TT0000350; • 1 ♀ (SEHU), Osima, Penins., Sengen-dake; 4–6.IX.1970, M. Suwa leg., TT0000348; • 2 ♀ (SEHU), Hakodate, Hakodate-yama, 20.VII.2015, H. Takizawa leg., H. Takizawa Coll., SEHU JAPAN, 2012 [typed on a green card], TT0000336, 337; Aomori Pref.: • 1 ♀ (SEHU), Zyuniko, 22.VI.1970, A. Abe leg, TT0000335; • 1 ♀ (SEHU), Shitsukari, Higashi-dôri vill., 13.VI.2003, Satoshi Araki leg., TT0000349.

#### Distribution

(of *Ch. angusticollis* treated as Morphological group A, B and C: Unit A-1, A-2, B-1, B-2, C-1, C-2 and C-3). Japan: Hokkaido, Honshu (Aomori Pref.).

#### Remarks.

*Ch. angusticollis* is most likely described from a monotype of a female. Species of subgenus Apterosoma are difficult to identify from external traits, and since the syntype is a single female, it is difficult to determine which units in the *Ch. angusticollis* species complex in northern Japan can be assigned to the name “*Ch. angusticollis*”. Each unit can be identified by their male genitalia, hind wings, distribution area and in some cases, coloration, elytral punctation, and body size. Males of most units can be reliably identified by the median lobes of their genitalia, while females can only be identified by the shape of their hind wings.

Therefore, we attempted to identify them through the following two methods:

1. Estimation based on type locality: As we mentioned above, Motschulsky (1861) recorded only “JAPAN” as the type locality in the original description, but according to Savitsky (2020), the species described by Motschulsky (1861), including *Ch. angusticollis*, were collected in the environs of Hakodate city (Oshima Dist., in the south of Hokkaido Isl.), or in the northern part of Honshu Isl. between Tokyo city and Tsugaru Strait by Mrs E.S. Goshkevitch in 1858 or earlier. The units distributed in the above-mentioned area are Unit B-1, Unit C-1 and Unit D, but Unit D is unlikely to be the relevant unit because it is only distributed in narrow regions of high altitude.
2. Checking of the syntype’s external traits: First, we examined the elytral surfaces of the syntype and found microscopic surface structure. The units displaying this microscopic surface structure on the elytra in the female are morphological groups A, B, C, and EI: Units A-1, A-2, B-1, B-2, C-1, C-2, C-3, EI-1 and EI-2 (Saitoh et al. 2008; Saitoh 2010, 2012).

**Figure 3.**
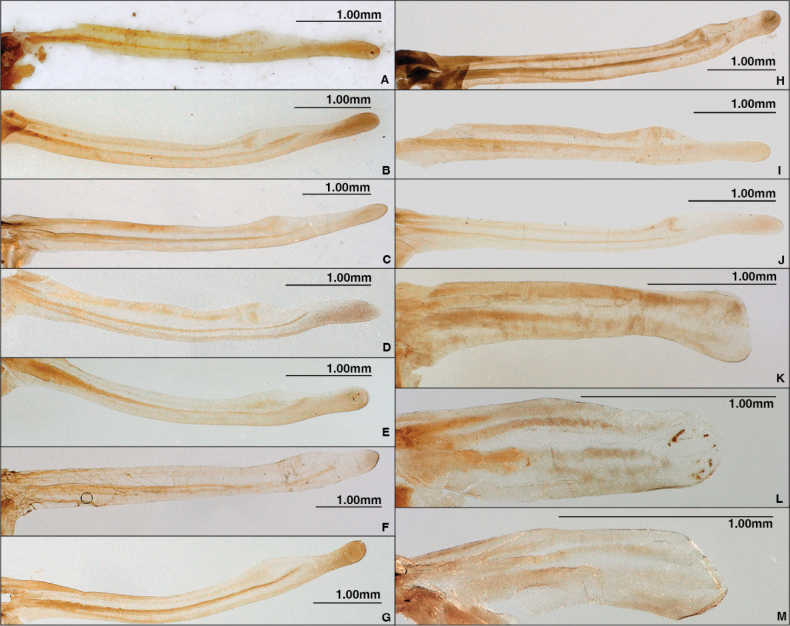
Hind wing of *Chrysolina angusticollis*, females (**A-J**) and *Chrysolina aino*, males (**I-K**). **A.** Syntype; **B–D.** Unit C-1. **B.** TT0000333; **C.** TT0000337; **D.** TT0000367; **E–H.** Unit B-1. **E.** TT0000335; **F.** TT0000368; **G.** TT0000342; **H.** TT0000369; **I, J.** Syntypes of *Chrysomela japana*. **I.** Fig. 4G–I; **J.** Fig. 4D–F; **K.** Holotype; **L.** Paratype (0000002423); **M.** Unit EII-2 (TT0000338). Photographs by Takuya Takemoto.

Next, we checked the shape of the hind wing of the syntype, which is expanded apically, pinched before the tip and expanded 2/5 length from the tip (Fig. 3A). In *Ch. angusticollis* species complex in northern Japan, only units B-1, C-1, 2, 3 and D exhibit a hindwing shape that is pinched before the tip. We examined individuals with this hindwing shape that were found in the vicinity of the collection site, estimated using method 1 and found individuals with hindwings similar to this hindwing shape in several localities in Hokkaido and Honshu. In these units, we found some variation in hind wing shape, the tip of which tended to be shorter in Unit B-1 and longer in Unit C-1 and in the syntype. Based on the above results, we have determined that syntype is very likely the equivalent of Unit C-1.

Mochizuki and Tsunekawa (1937) recorded *Ch. angusticollis* from Korea for the first time, but this record is excluded here because it is not an appropriate distribution, assuming that *Ch. angusticollis* is treated as Morphological group A, B and C: Units A-1, A-2, B-1, B-2, C-1, C-2 and C-3, as indicated above. Krivolutskaya and Medvedev (1966) recorded this species from Kunashiri Isl. for the first time. Later on, Dubeshko and Medvedev (1989) recorded this species from “Amur region” and “South Kuriles”. Subsequently the specimen from Kunashiri Isl. was identified by Bieńkowski (2007) as *Ch. porosirensis*. The record from the Amur region was first made by Jacobson (1893), although later he reexamined these specimens and described them as a new species Chrysolina (Anopachys) lineigera (Jacobson, 1901). Therefore, *Ch. angusticollis* is currently distributed only in Japan. According to Bieńkowski (2001), there is a specimen collected from Honshu, Yokohama as “Additional specimens”, but the record of *Ch. angusticollis* from Yokohama should be confirmed because we have not been able to find any other records or identify any specimens from that locality.

**Figure 4.**
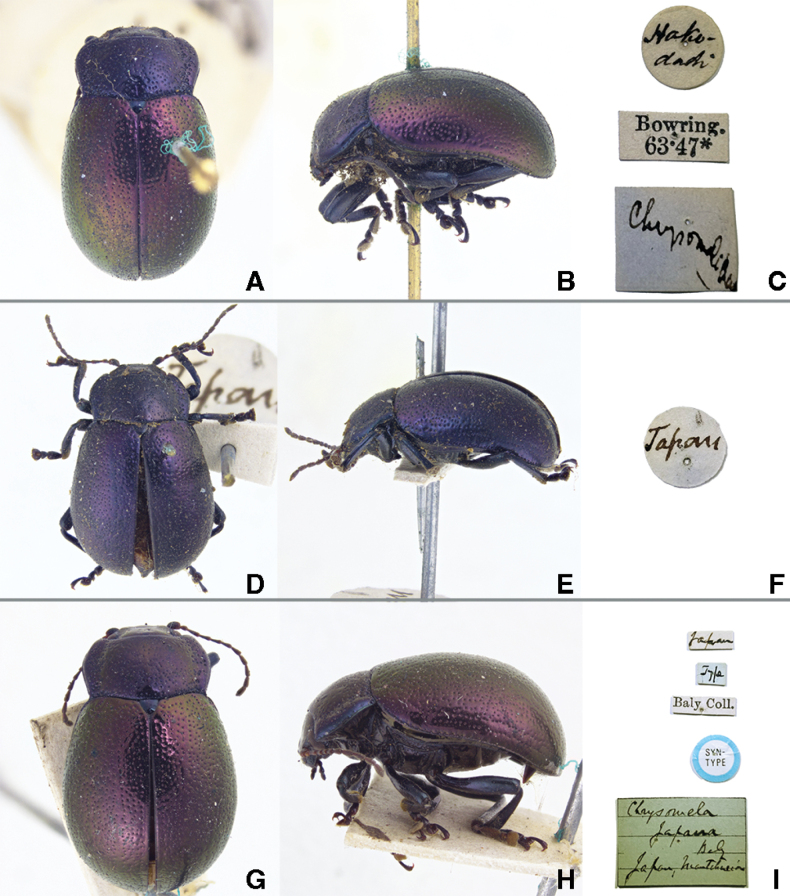
*Chrysomela japana* Baly, 1874, syntypes. **A.** Male habitus, dorsal view; **B.** Female habitus, lateral view; **C.** Labels; **D.** Female habitus, dorsal view; **E.** Male habitus, lateral view; **F.** Labels; **G.** Female habitus, dorsal view; **H.** Female habitus, lateral view; **I.** Labels. Photographs by Takuya Takemoto.

We also checked the syntypes of *Chrysomela japana* Baly, 1874, the only junior synonym of *Ch. angusticollis*, and which units they correspond to. There are three specimens (one male and two females) of the syntypes of *Chrysomela japana* in BMNH, and according to Baly (1874), the first specimen (Fig. 4A–C) was collected from “Hakodadi” by Mr Whitely in the Lewis Collection, the second specimen (Fig. 4D–F) was collected from “Japan” by Mr Moor in the Baly collection, and the third specimen (Fig. 4G–I) was collected from “Manchuria” by Mr Bowring. If the data on these labels is correct, the first specimens should correspond to Unit C-1, but the second specimen is in a similar situation to the syntype of *Ch. angusticollis*, and the shape of the hindwing must be confirmed. As for the third specimen, it could possibly be the only record from China.

We checked the shape of the hind wing of the second (Fig. 4D–F) and third (Fig. 4G–I) syntypes, and found that each is expanded apically, pinched before the tip, and expanded at 2/5 length from the tip (Fig. 3I, J) as seen in Unit C-1

We checked the elytral surfaces of these syntypes and found microscopic surface structure. Although we cannot state here whether *Ch. angusticollis* is distributed in China, we found that at least all syntypes of *Chrysomela japana* are extremely similar to Unit C-1.

### ﻿ Chrysolina aino

Taxon classificationAnimaliaColeopteraChrysomelidae

Takizawa, 1970

0C1A39B5-09A7-5F0D-A92A-C68CC95A10D4

Figs 3K–M, 5, 6

Chrysolina aino Takizawa, 1970: 117 (Japan: Hokkaido, Sapporo, Hoheikyo, holotype and paratypes in SEHU).

#### Type material examined.

***Holotype*** • ♂ (SEHU), Sapporo / Hokkaido / 17.VIII.1966 / H. Takizawa / Hoheikyo, leg. T. Kocha (typed on the back of the label) // Holo-type / Chrysolina ainu / Takizawa [typed on a red card] // HOLOTYPE / Appended label by / ÔHARA, INARI, KANBE / SUZUKI and HIRONAGA / 2006 // 000000261 / Sys. Ent / Hokkaido Univ. / Japan [SEHU].

Measurements of holotype. HW 2.33 mm; PEL 7.80 mm; EL 5.99 mm; EW 4.85 mm; APW 2.63 mm; PL 2.25 mm; MPW 3.73 mm; PPW 3.42 mm.

***Paratypes***, **Japan.** Hokkaido: • 1 ♀ (SEHU), Sapporo/ 4.IX.1965 / H. Takizawa / Zyozankei (typed on the back of the label) // Paratype / Chrysolina ainu / Takizawa [typed on a pink card]; • 8 ♂ (SEHU), Sapporo/ 10.VII.1966 / H. Takizawa / Hoheikyo, leg. T. Kocha (typed on the back of the label) // Paratype / Chrysolina ainu / Takizawa [typed on pink card] // 0000002428 – 0000002435 / Sys. Ent / Hokkaido Univ. / Japan [SEHU] // PARATYPE / Appended label by / ÔHARA, INARI, KANBE / SUZUKI and HIRONAGA / 2007; • 2 ♀ (SEHU), same labels but 17.VIII.1966 and 0000002421, 0000002422; • 1 ♂ (SEHU), Nukabira / 17.VI.1966 / H. Takizawa // Paratype / Chrysolina ainu / Takizawa // PARATYPE / Appended label by / ÔHARA, INARI, KANBE / SUZUKI and HIRONAGA / 2007 // 0000002423 / Sys. Ent / Hokkaido Univ. / Japan [SEHU]; • 1 ♀ (SEHU), same labels but collected from Sapporo and 0000002424; • 1 ♂ (SEHU), Sapporo/ 9.VI.1966 / H. Takizawa / Hoheikyo (typed on the back of the label) // Paratype / Chrysolina ainu / Takizawa [typed on a pink card] // 0000002425 / Sys. Ent / Hokkaido Univ. / Japan [SEHU] // PARATYPE / Appended label by / ÔHARA, INARI, KANBE / SUZUKI and HIRONAGA; • 1 ♂ (SEHU), same labels but 18.VIII.1966 and 0000002426; • 1 ♂ (SEHU), Sapporo / Hokkaido / 4.IX.1965 / H. Takizawa / Zenibako-toge (typed on the back of the label) // Paratype / Chrysolina ainu / Takizawa [typed on pink card] // 0000002427 / Sys. Ent / Hokkaido Univ. / Japan [SEHU] // PARATYPE / Appended label by / ÔHARA, INARI, KANBE / SUZUKI and HIRONAGA / 2007; • 1 ♀ (SEHU), Sapporo/ 24.VIII.1966 / H. Takizawa / Mt. Sora-numa (typed on the back of the label) // Paratype [typed on a blue card] // PARATYPE / Appended label by / ÔHARA, INARI, KANBE / SUZUKI and HIRONAGA / 2007 // 0000003028 / Sys. Ent / Hokkaido Univ. / Japan [SEHU]; • 1 ♂ (SEHU), same labels but 7.VII.1965 and 0000003030; • 1 ♀ (SEHU), Sapporo/ 8.VI.1966 / H. Takizawa / Hoheikyo (typed on the back of the label) // Paratype [typed on a blue card] // PARATYPE / Appended label by / ÔHARA, INARI, KANBE / SUZUKI and HIRONAGA / 2007 // 0000003029 / Sys. Ent / Hokkaido Univ. / Japan [SEHU]; • 1 ♂, 3 ♀ (SEHU), Sapporo / 14.VII.1965 / H. Takizawa / Zyozankei (typed on the back of the label) // Paratype [typed on a blue card] // PARATYPE / Appended label by / ÔHARA, INARI, KANBE / SUZUKI and HIRONAGA / 2007 // 0000003031 – 3033, 0000003038 / Sys. Ent / Hokkaido Univ. / Japan [SEHU]; • 1 ♀ (SEHU), same labels but 3.VIII.1965 and 0000003039; • 1 ♂, 1 ♀ (SEHU), Sapporo / 10.VII.1966 / H. Takizawa / Hokeikyo (typed on the back of the label) // Paratype [typed on a blue card] // PARATYPE / Appended label by / ÔHARA, INARI, KANBE / SUZUKI and HIRONAGA / 2007 // 0000003040, 0000003041 / Sys. Ent / Hokkaido Univ. / Japan [SEHU]; • 2 ♂ (SEHU), Sapporo / 30.VI.1966 / H. Takizawa / Mt. Moiwa (typed on the back of the label) // Paratype [typed on a blue card] // PARATYPE / Appended label by / ÔHARA, INARI, KANBE / SUZUKI and HIRONAGA / 2007 // 0000003042, 0000003043 / Sys. Ent / Hokkaido Univ. / Japan [SEHU]; • 1 ♀ (SEHU), Sapporo / 19.VII.1965 / H. Takizawa / Zenibako-toge (typed on the back of the label) // Paratype [typed on a blue card] // PARATYPE / Appended label by / ÔHARA, INARI, KANBE / SUZUKI and HIRONAGA / 2007 // 0000003044 / Sys. Ent / Hokkaido Univ. / Japan [SEHU]; • 1 ♂ (SEHU), same labels but 7.VII.1965 and 0000003045.

#### Other material examined.

**Japan.** Hokkaido: • 1 ♂ (SEHU), Soranuma-dake, VI.1977, emergence (Bred.), NR. 458., TT0000338; • 1 ♂ (SEHU), Tokachi, Nukabira, 11-VII.1961, I. Miyagi leg., TT0000346; • 1 ♀ (SEHU), Nukabira, 3-VIII. 1949, C. Watanabe leg. (typed on the back of the label), TT0000347.

#### Distribution.

Japan: Hokkaido.

#### Remarks.

**Figure 5.**
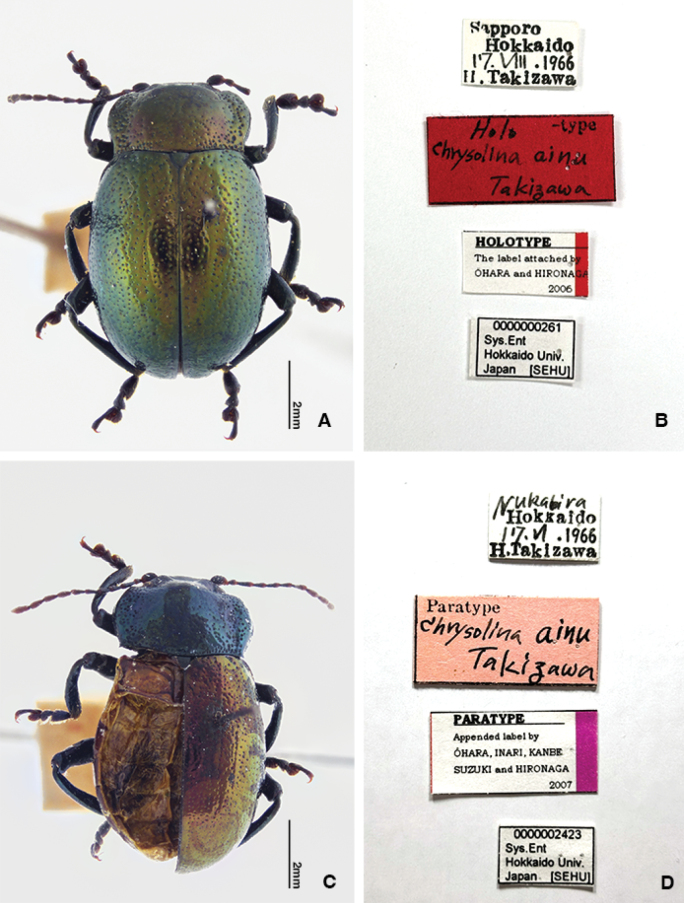
*Chrysolina aino* Takizawa, 1970. **A, B.** Holotype. **A.** Habitus, dorsal view; **B.** Labels; **C, D.** Paratype (0000002423). **C.** Habitus, dorsal view; **D.** Labels. Photographs by Takuya Takemoto.

As mentioned above, at present, *Ch. aino* is treated as Morphological group EII and EIII: Unit EII-1, EII-2, EIII-1 and EIII-2. Considering the distribution range, two paratype specimens collected at “Mt. Sora-numa” (0000003028, 0000003030) ought to be considered Unit EII-2, but after checking external traits, they were found to be Unit EIII-1. Both holotype and paratype correspond to Unit EIII-1, but one of the paratypes, (0000002423) collected in Nukabira has different hind wings and median lobe of the male genitalia. Nukadaira is located in the northern part of the Tokachi region of Hokkaido, and Unit F (*Ch. porosirensis*) is distributed here. However, this paratype differs from Unit F in external traits such as the shape of the median lobe and hind wings. The apex of the median lobe of this paratype gradual expands toward the apex in dorsal view (Fig. 5C, D), the hind wing is remarkably degenerated and furnished with four veins (Fig. 3J). The pronotum is greenish blue with a sheen and the elytron is yellowish orange with a sheen (Fig. 4C). These features are similar to Unit EII-2 distributed from the southern area of Ishikari District to the western part of Iburi District. Since it is unlikely that this unit has an exclave distribution, we suspected that it was mislabeled. However, we found two other specimens (TT0000346, 347) collected at Nukabira on different dates by different collectors that are very similar to this paratype. This indicates that the label of the paratype is not mistyped and there is a possibility that Unit EII-2 or another similar unit is distributed in Nukabira, but the number of specimens is too small to properly characterize the identity these populations.

**Figure 6.**
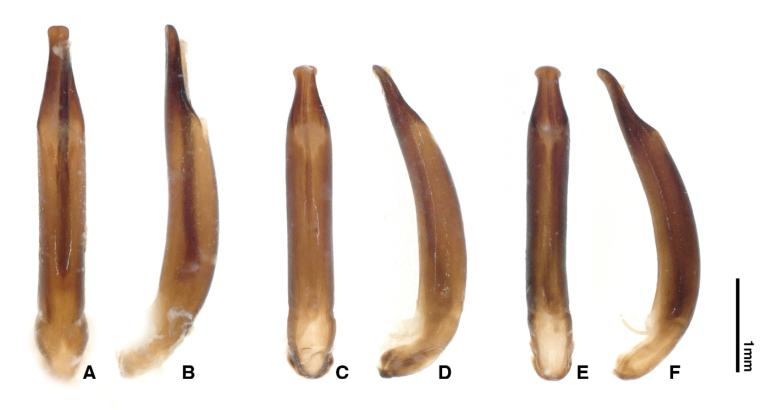
Median lobes of *Chrysolina aino*. **A, B.** Holotype. **A.** Dorsal view; **B.** Lateral view; **C, D.** Paratype (0000002423). **C.** Dorsal view; **D.** Lateral view; **E, F.** Unit EII-2 (TT0000338). **E.** Dorsal view; **F.** Lateral view. Photographs by Takuya Takemoto.

All type labels of this species are mistyped as *Chrysolina* “*ainu*”, instead of *Chrysolina aino*. In the original description, the species epithet was given as “*aino*”, and subsequent literature has consistently used this spelling. Upon inquiry, Dr Takizawa, the author of the original description, confirmed that the spelling “*ainu*” found on the type labels was a handwritten mistake, and that *Chrysolina aino* is the correct and valid name.

In Bieńkowski (2007), *Ch. aino* is recorded from Korea for the first time, but this record is excluded here because it is not an appropriate distribution assuming that *Ch. aino* is treated as Morphological group EII and EIII: EII-1, EII-2, EIII-1 and EIII-2, as indicated above.

According to Bieńkowski (2001), there are two specimens collected from Honshu, Kobe as “Additional specimens”. From examining them, the habitus and genitalia agree with Unit C-1. This record, as well as the record of *Ch. angusticollis* from Yokohama, need to be re-examined because no other records or other specimens have been found, and we will refrain from recording it here as a distribution.

### ﻿ Chrysolina porosirensis

Taxon classificationAnimaliaColeopteraChrysomelidae

Takizawa, 1970

9FFFD7D4-FD7C-5F6E-8F63-4BD47E8CC0B9

Fig. 7

Chrysolina porosirensis Takizawa, 1970: 120 (Japan: Hokkaido, Niikappu, Nanatsu-numa Cirque, holotype and paratypes in SEHU).
Chrysolina porosinensis (sic): Bourdonné and Doguet (1991): 56.

#### Type material examined.

***Holotype*** • ♂ (SEHU), Nanatunuma / Hidaka Mts. / 10.VII.1965 / Umezawa // Holo -type / Chrysolina / porosirensis / Takizawa [typed on a red label] // HOLOTYPE / The label attached by / ÔHARA and HIRONAGA / 2006 // 0000000264 / Sys. Ent / Hokkaido Univ. / Japan [SEHU].

**Figure 7.**
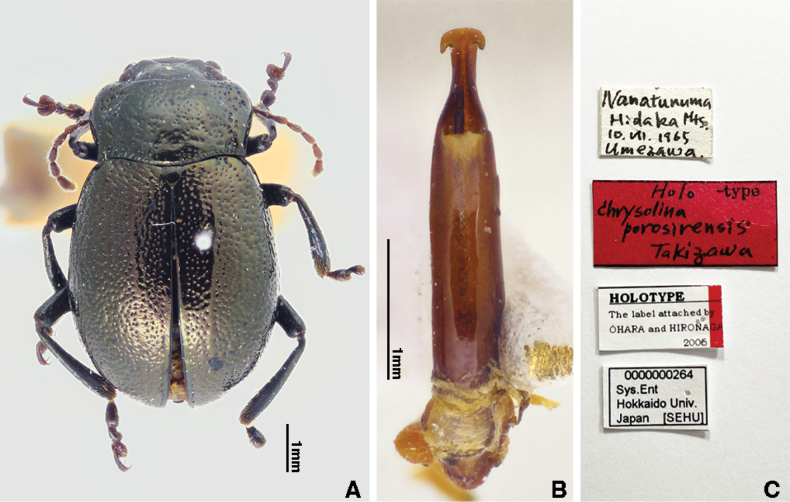
*Chrysolina porosirensis* Takizawa, 1970, holotype. **A.** Habitus, dorsal view; **B.** Median lobe, dorsal view; **C.** Labels. Photographs by Takuya Takemoto.

Measurements of Holotype. HW 2.21 mm; PEL 6.33 mm; EL 4.69 mm; EW 3.92 mm; APW 2.42 mm; PL 1.85 mm; MPW 3.39 mm; PPW 3.04 mm.

***Paratypes*** • 8 ♂, 1 ♀ (SEHU), Mt. Porosiri, Mts. Hidaka / Hokkaido, 21.Vii.1967 / Kumata, Kocha, Ueda et. // Paratype / Chrysolina / porosirensis / Takizawa // PARATYPE / Appended label by / ÔHARA, INARI, KANBE / SUZUKI and HIRONAGA / 2007 // 0000002409, 2410, 2413, 2415–2420 / Sys. Ent / Hokkaido Univ. / Japan [SEHU]; • 1 ♀ (SEHU), Nanatunuma / Hidaka Mts., / 10.VII.1965 / Umegawa //Paratype / C. porosiren- / sis, Taki. // 0000002414 / Sys. Ent / Hokkaido Univ. / Japan [SEHU] // PARATYPE / Appended label by / ÔHARA, INARI, KANBE / SUZUKI and HIRONAGA / 2007; • 1 ♂ (SEHU), Nukabira / Hokkaido / 18.VI.1966 / H. Takizawa // (フキ) // Paratype / Chrysolina porosirensis / Takizawa [typed on a pink card] // PARATYPE / Appended label by / ÔHARA, INARI, KANBE / SUZUKI and HIRONAGA / 2007 // 0000002411 / Sys. Ent / Hokkaido Univ. / Japan [SEHU]; • 1 ♂ (SEHU), Daiseuzan / Hokkaido / 27.VII.1965 / T. Kocha / Mt. Hakuun dake (typed on the back of the label) // Paratype / Chrysolina porosirensis / Takizawa [typed on a pink card] // 0000002412 / Sys. Ent / Hokkaido Univ. / Japan [SEHU] / ARATYPE / Appended label by / ÔHARA, INARI, KANBE / SUZUKI and HIRONAGA / 2007.

#### Distribution.

Kunashiri Isl., Hokkaido.

## ﻿Conclusion

In this study, *Ch. angusticollis* was confirmed to correspond to Morphological Group C, Unit C-1, as established by Saitoh (2012). Furthermore, examination of the three syntypes of *Chrysomela japana* Baly, 1874, which have been treated as a synonym of *Ch. angusticollis*, revealed that all specimens correspond to Unit C-1, and thus there is no reason to reject its synonymy with *Ch. angusticollis*. As for *Ch. aino* and *Ch. porosirensis*, their correspondence to the units shown in Table 1 was confirmed to be accurate, as stated by Saitoh (2012). However, as indicated in the table, the subgenus Apterosoma appears to include many more species or subspecies, and a comprehensive systematic revision is necessary.

## Supplementary Material

XML Treatment for Apterosoma

XML Treatment for Chrysolina angusticollis

XML Treatment for Chrysolina aino

XML Treatment for Chrysolina porosirensis

## ﻿Additional information

### Author ORCIDs

Takuya Takemoto https://orcid.org/0009-0004-9562-6897

### Data availability

All of the data that support the findings of this study are available in the main text.
